# The framing of time-dependent machine learning models improves risk estimation among young individuals with acute coronary syndromes

**DOI:** 10.1038/s41598-023-27776-0

**Published:** 2023-01-19

**Authors:** Luiz Sérgio Fernandes de Carvalho, Gustavo Alexim, Ana Claudia Cavalcante Nogueira, Marta Duran Fernandez, Tito Barbosa Rezende, Sandra Avila, Ricardo Torres Bispo Reis, Alexandre Anderson Munhoz Soares, Andrei Carvalho Sposito

**Affiliations:** 1 Laboratory of Data for Quality of Care and Outcomes Research (LaDa:QCOR), Catholic University of Brasília, Taguatinga Sul, Brasília, DF 71966-700 Brazil; 2 Aramari Apo Institute for Education and Clinical Research, Brasília, DF Brazil; 3 Clarity Healthcare Intelligence, Jundiaí, SP Brazil; 4 Faculty of Medicine, University of Brasília, Brasília, DF Brazil; 5 Escola Superior de Ciências da Saúde, Brasília, DF Brazil; 6 Faculty of Electrical Engineering and Computation, State University of Campinas (UNICAMP), Campinas, SP Brazil; 7 Institute of Computing, UNICAMP, Campinas, SP Brazil; 8 Department of Statistics, University of Brasília, Brasília, DF Brazil; 9 Cardiology Department, UNICAMP, Campinas, SP Brazil

**Keywords:** Cardiology, Medical research, Epidemiology, Outcomes research

## Abstract

Acute coronary syndrome (ACS) is a common cause of death in individuals older than 55 years. Although younger individuals are less frequently seen with ACS, this clinical event has increasing incidence trends, shows high recurrence rates and triggers considerable economic burden. Young individuals with ACS (yACS) are usually underrepresented and show idiosyncratic epidemiologic features compared to older subjects. These differences may justify why available risk prediction models usually penalize yACS with higher false positive rates compared to older subjects. We hypothesized that exploring temporal *framing structures* such as prediction time, observation windows and subgroup-specific prediction, could improve time-dependent prediction metrics. Among individuals who have experienced ACS (n_*global_cohort*_ = 6341 and n_yACS_ = 2242), the predictive accuracy for adverse clinical events was optimized by using specific rules for yACS and splitting short-term and long-term prediction windows, leading to the detection of 80% of events, compared to 69% by using a rule designed for the global cohort.

## Introduction

In the last four decades, a major concern has arisen from the progressive increase in the incidence rates of acute coronary syndrome (ACS) among young individuals (yACS, i.e. before 55 years of age)^1,2^ and the high recurrence rate of these events^3^. In addition to amplifying ACS-related reduction in quality of life and life expectancy, yACS also carries a heavy economic burden by reducing work capacity in early adulthood^3–5^.

Young individuals with ACS are more likely to be men, smokers, obese, sedentary or to present familial-combined hyperlipidemia, and they more frequently consume cocaine or androgenic anabolic steroids than older ACS patients^6,7^. Furthermore, compared with older individuals, those with yACS have a higher proportion of traditional cardiovascular risk factors out of control^7,8^.

This setting of frequent uncontrolled risk factor among yACS triggers a relevant economic impact^8^. However, there is no easy path to prevent recurrent events among yACS as adherence to risk factor modification measures is typically low in real-world conditions^8^. Strategies to improve adherence with intensified multifactorial intervention effectively reduced the risk of death and cardiovascular events^9,10^, but these approaches are economically unfeasible in a large population. On the other hand, a health policy tied to risk prediction and multifactorial intervention directed to high-risk individuals could represent a cost-effective solution^5^. Despite the clear distinction in the recurrence of coronary events, the prevalence of risk factors and economic burden among yACS, effective risk prediction tools specific to individuals with yACS remain an unmet need.

In clinical research, risk prediction tools infrequently explore the role of the observation window, i.e., the moment when predictors are captured, and the forecast window, i.e., the period from which the event is surveyed or sampled. As reviewed recently, the match between the clinical problem and these temporal *framing structures* is essential for high-quality predictive models^11^. This indicates that some *“acute phase”* information captured during ACS hospitalization may be useful to predict short-term outcomes but may not be useful to predict long-term outcomes^12,13^. We therefore hypothesized that splitting predictive rules into two (short- and long-term) would allow more accurate risk prediction.

While for short-term outcomes it is commonly accepted that binary classification rules are reasonable, for instance *major adverse cardiovascular events* [MACE] vs non-MACE^12^, long-term predictive models need to consider time-to-event with competing events to minimize censoring bias^14,15^. To develop and validate models, we used interpretable and *state-of-the-art* algorithms for tabular data to predict *in-hospital* outcomes^16^ and used survival analysis with competing risks^14^ to predict long-term clinical events in a large cohort of yACS individuals. Furthermore, we studied the differences in risk factors and optimal prediction rules for yACS compared to ACS in older subjects.

## Results

The study population had a mean age of 48 ± 6 years, and 66% were male. Table 1 depicts the characteristics of yACS subjects (n = 2242) compared to older individuals with ACS (n = 4099). A total of 170 deaths (11.4 per 1000 patients-years), 132 STEMI (8.8 per 1000 patients-years) and 421 NSTEMI (28.2 per 1000 patients-years) occurred after a median follow-up of 6.67 years (95% confidence interval [CI] of 5.59–7.24) among yACS individuals. As described in Supplemental Table S1, *in-hospital* MACEs occurred in 180 individuals, and postdischarge MACEs occurred in 454 subjects with yACS. Among subjects older than 55 years old, *in-hospital* MACEs occurred in 493 individuals, and postdischarge MACEs occurred in 881.

**Table 1:** Baseline characteristics and clinical outcomes of individuals with premature acute coronary syndrome (ACS, ≤ 55 years old) and older subjects with ACS (> 55 years old).

	ACS > 55 years-old	ACS ≤ 55 years-old	*p*
n	4099	2242	
Age (mean (SD))	68.47 (8.11)	47.63 (5.84)	< 0.001
Male gender (%)	60.8	66.1	< 0.001
**Diagnoses**			
Index diagnosis (%)			< 0.001
STEMI	60.9	74.0	
NSTEMI	20.1	15.5	
UA	19.0	10.5	
T2DM (%)	34.0	27.5	< 0.001
T2DM on insulin (%)	9.4	8.1	0.177
Smokers (%)	34.7	41.2	< 0.001
Dyslipidemia (%)	20.2	19.3	0.550
Hypertension (%)	74.7	66.9	< 0.001
Obesity (%)	5.2	7.9	0.001
Family history of prCAD (%)	9.8	16.6	< 0.001
Prior ethylic habit (%)	9.7	14.1	< 0.001
Prior drug abuse (%)	1.3	6.9	< 0.001
Prior AMI (%)	8.5	7.1	< 0.001
Prior stroke (%)	4.1	2.2	0.003
Prior PAD (%)	4.3	2.9	0.025
Prior CKD (%)	8.5	2.5	< 0.001
Prior PCI (%)	8.9	6.2	0.002
Prior CABG (%)	5.6	3.7	0.007
Prior cocaine abuse (%)	0.1	1.3	< 0.001
Prior marijuana abuse (%)	0.1	0.8	0.027
Atrial fibrillation (%)	3.7	0.8	< 0.001
**Drugs prescribed at discharge**			
Nitrate (%)	45.1	42.5	0.123
Statin (%)	83.0	81.9	0.385
Betablockers (%)	64.9	66.6	0.293
ARB or ACEi (%)	58.4	61.7	< 0.001
CCB (%)	20.0	18.5	0.278
ASA (%)	91.0	89.4	0.153
Clopidogrel (%)	62.5	64.4	0.244
Prasugrel (%)	21.8	22.0	0.941
Ticagrelor (%)	3.5	4.0	0.422
Anticoagulant (%)	3.3	3.5	0.816
Spironolactone (%)	11.7	7.9	< 0.001
Furosemide (%)	15.2	9.4	< 0.001
**Severe coronary artery lesions**			
1-vessel with proximal LAD (%)	4.8	8.8	< 0.001
1-vessel with LAD (%)	8.5	9.9	< 0.001
1-vessel with RCA (%)	10.4	12.6	< 0.001
2-vessels without LAD (%)	13.1	11.2	0.133
3-vessels with LAD (%)	13.3	8.9	< 0.001
3-vessels without LAD (%)	16.1	10.6	< 0.001
3-vessels with LCA and LAD (%)	1.8	0.4	< 0.001
3-vessels with LCA/without LAD (%)	1.2	0.3	0.007
**PCI—index coronarography**			
LAD (%)	13.5	18.5	< 0.001
MINOCA (%)	2.4	1.7	0.223
Number of new stents (mean (SD))	1.38 (0.72)	1.44 (0.70)	< 0.001
**Echocardiography**			
Apical dyskinesia	3.5	1.1	< 0.001
Apical akinesia	29.6	23.7	< 0.001
LV function at 3rd to 5th day (%)			0.005
> 45%	22.3	24.4	
< 45%	77.7	75.6	
**Admission metrics (STEMI individuals only; n = 4042)**			
Cardiac arrest before admission (%)	2.3	0.3	< 0.001
Time pain-primary hospital, minutes (mean (SD))	164.51 (142.39)	153.66 (131.44)	0.043
Time door-needle, minutes (median [IQR])	70.00 [43.00, 120.00]	68.00 [43.00, 110.50]	0.269
Time pain-needle, minutes (median [IQR])	225.00 [150, 335]	210.00 [140, 315]	0.005
Time tnk-coronarography, minutes (mean (SD))	1195.82 (1269.01)	1270.69 (1137.35)	0.114
Coronarography duration, minutes (median [IQR])	55.00 [40.00, 75.00]	50.00 [40.00, 65.00]	< 0.001
Pharmacoinvasive strategy (%)	88.9	90.2	0.698
Primary PCI (%)	11.1	9.8	0.452
SBP at admission, mmHg (mean (SD))	121.86 (26.01)	143.43 (25.22)	< 0.001
DBP at admission, mmHg (mean (SD))	75.46 (17.19)	88.18 (17.21)	< 0.001
HR at admission, beats/minute (mean (SD))	81.00 (19.47)	77.40 (15.44)	< 0.001
**Killip score (%)**			< 0.001
I	43.5	84.0	
II	25.9	14.2	
III	16.2	0.9	
IV	14.4	0.9	
TIMI flow pre-PCI (mean (SD))	2.05 (1.22)	2.20 (1.18)	0.003
TIMI flow post-PCI (mean (SD))	2.58 (0.81)	2.69 (0.75)	0.002
MBG pre-PCI (mean (SD))	1.61 (1.45)	1.84 (1.42)	< 0.001
MBG post-PCI (mean (SD))	1.86 (1.35)	2.18 (1.22)	< 0.001
**Clinical scores**			
TIMI score (mean (SD))	5.15 (2.35)	2.46 (1.56)	< 0.001
GRACE in-hospital death (mean (SD))	150.80 (32.75)	89.47 (18.19)	< 0.001
GRACE score (6 months) (mean (SD))	141.49 (24.28)	91.61 (15.96)	< 0.001
CRUSADE (mean (SD))	35.75 (13.87)	19.44 (11.29)	< 0.001
**Laboratory exams**			
Troponin (peak) (mean (SD))	9999 (10,399)	6838 (6465)	< 0.001
Glycemia, mg/dL (mean (SD))	159.51 (81.38)	137.86 (62.80)	< 0.001
HbA1c, % (mean (SD))	6.87 (2.03)	6.63 (2.01)	0.014
Total cholesterol, mg/dL (mean (SD))	194.74 (48.58)	204.71 (46.58)	< 0.001
LDL-cholesterol, mg/dL (mean (SD))	122.82 (39.59)	132.19 (40.23)	< 0.001
Triglycerides, mg/dL (mean (SD))	152.54 (133.41)	166.06 (118.62)	0.009
Creatinine clearance, ml/min/1.73m2 (mean (SD))	74.52 (28.94)	107.10 (33.94)	< 0.001
BMI, kg/m2 (mean (SD))	26.65 (4.57)	27.15 (4.51)	0.005
**Clinical outcomes (considering competing risks)**			
In-hospital deaths (%)	5.7	3.9	< 0.001
Post-discharge deaths (%)	9.6	4	< 0.001
Global deaths (%)	15.3	7.6	< 0.001
Global CV deaths (%)	5.2	3.5	0.018
Global non-CV deaths (%)	10.1	4.0	< 0.001
MI during follow-up (%)			< 0.001
STEMI	12.8	5.9	
NSTEMI	15.5	18.8	
Follow-up time, days (median [IQR])	2331 [2030, 2625]	2436 [2039, 2644]	< 0.001
CABG (in-hospital) (%)	4.7	2.8	0.017
CABG during long-term follow-up (%)	13.3	10.9	0.026

*CABG* Coronary artery bypass graft, *CKD* Chronic kidney disease, *GFR* Glomerular filtration rate (CKD-EPI), *GPIIbIIIa* Glycoprotein IIbIIIa, *HbA1c* Glycosylated hemoglobin, *LDL-C* Low-density lipoprotein cholesterol, *LV* Left ventricle, *BMI* Body mass index, *NSTEMI* Non-ST-elevation myocardial infarction, *PCI* Percutaneous coronary intervention, *STEMI* ST-elevation myocardial infarction, *SBP* Systolic blood pressure, *DBP* Diastolic blood pressure, *HR* Heart rate, *CV* Cardiovascular.

Younger subjects were more frequently smokers and obese and had a more frequent family history of premature CAD and personal history of alcohol or cocaine use (Table 1). Although type 2 diabetes mellitus (T2DM) was less frequently found and the global mean for HbA1c was lower among yACS, among subjects with T2DM, those with yACS presented a higher HbA1c (9.08 ± 0.92%) than their older counterparts (8.12 ± 1.09%; *p* < 0.0001). Younger subjects were more frequently admitted due to STEMI, but the severity was generally lower than that in older subjects with ACS, as cardiac arrest before admission and Killip scores III or IV were less frequent. As expected, the burden of coronary artery disease was also lower among yACS individuals (Table 1).

As shown, the yACS subgroup shows a highly distinctive epidemiologic profile compared with older ACS subjects. Therefore, we explored which are the key risk factors for MACEs in the short- and long-term among the two subgroups and found different patterns.

### Short-term MACE

STWm has shown that there are significant differences in key predictors and model accuracy both by using stepwise LR (sLR) and more complex predictive algorithms. While the *sLR* model within the global cohort (training/validation with n = 4439) showed an *accuracy* in the yACS test set (n = 673) of 0.82 (95% CI of 0.79–0.84) and *a C-statistic* of 0.79 (95% CI of 0.77–0.81), an sLR developed specifically within the yACS individuals showed a significantly higher *C-statistic* of 0.87 (95% CI of 0.85–0.89, *p* for *C-statistic* comparison < 0.001) in the yACS test set (Table 2). Supplementary Tables S2 and S3 show that the most important predictor variables in *sLR* to explain at least 90% of model variance were different in yACS and the global cohort. In yACS subjects, the *odds ratios* for MACE compared to the global cohort were higher for blood glycemia, prior chronic kidney disease (CKD), Killip class and syncope at ACS onset and lower for myocardial blush grade (MBG) and presence of dyskinesia (any wall). Increasing duration of catheterization (cath) was highly associated with MACEs in yACS and linked to intraprocedural complications such as coronary artery dissections (3.02% of yACS) and coronary rupture (0.1% of yACS). Late catheterization (12 h after symptom onset for STEMI and 24 h after symptom onset for UA/NSTEMI) was also an independent risk factor for MACE only in yACS.

**Table 2:** *Accuracy* and *C-statistics* for short-term window models in predicting *in-hospital* death or recurrent ischemic events in 2242 individuals with premature ACS (55 years old or younger), total number of events = 180 (*in-hospital* CV deaths = 39, and MI = 141).

	Accuracy (95% confidence interval)	C-statistics (95% confidence interval)
***Logistic regression* with GRACE score risk factors**		
Validation set (mean of 5-folds)	0.841 (0.819–0.866)	0.834 (0.819–0.866)
Test set (n = 673,75 events)	0.820 (0.784–0.856)	0.819 (0.782–0.853)
***Logistic regression* (top 20 predictors)**		
Validation set (mean of 5-folds)	0.889 (0.867–0.904)	0.883 (0.867–0.904)
Test set (n = 673,75 events)	0.880 (0.854–0.896)	0.872 (0.851–0.894)
***Random Forests* (top 20 predictors)**		
Validation set (mean of 5-folds)	0.901 (0.870–0.928)	0.908 (0.879–0.933)
Test set (n = 673,75 events)	0.892 (0.852–0.930)	0.888 (0.846–0.927)
***XGBoost* (top 20 predictors)**		
Validation set (mean of 5-folds)	0.894 (0.870–0.929)	0.898 (0.872–0.931)
Test set (n = 673,75 events)	0.876 (0.859–0.903)	0.861 (0.835–0.891)
***TabNet* (top 20 predictors)**		
Validation set (mean of 5-folds)	0.951 (0.924–0.979)	0.936 (0.904–0.955)
Test set (n = 673,75 events)	0.946 (0.917–0.975)	0.921 (0.889–0.953)

The *sLR* model trained in the yACS cohort performed as well as *the random forest* and *XGBoost* algorithms (*p* for *C-statistic* comparisons of 0.68 and 0.77, respectively), and *sLR* was superior to the GRACE score-based model (*p* = 0.031) (Table 2). However, with a *C-statistic* of 0.92 (95% CI 0.89–0.95), the *TabNet* algorithm trained in the yACS cohort was superior to sLR (*p* for *C-statistic* comparisons < 0.001) and superior to *TabNet* trained in the global cohort (C-statistic of 0.90 (95% CI 0.88–0.92), *p* for *C-statistic* comparisons 0.011).

**Figure 1:**
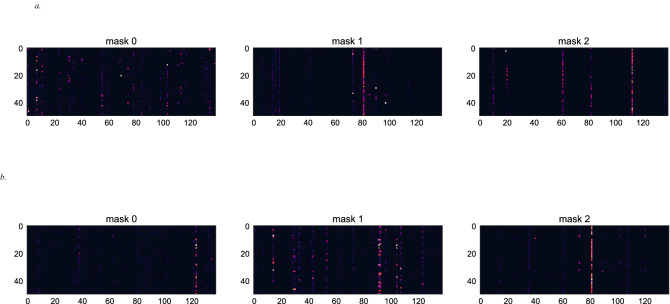
Feature importance masks (indicating feature selection at the *i*th step) and the aggregate feature importance mask (mask 0) showing the global instancewise feature selection on the global cohort (**a**) and young subjects with ACS (**b**). Brighter colors show higher values. Legend: The x axis represents each feature used in *B-CaRe:QCO* dataset; and the y axis represents the first 50 test samples. Features shown in vivid/bright colors were more intensely recruited, and features shown in dark colors were less intensely recruited. In the global cohort, 28 variables are recruited by *TabNet* algorithm, and 20 are responsible for 91% of the model variance. In the yACS cohort, 24 variables were recruited by *TabNet* algorithm, and 20 were responsible for 93% of the model variance.

**Figure 2:**
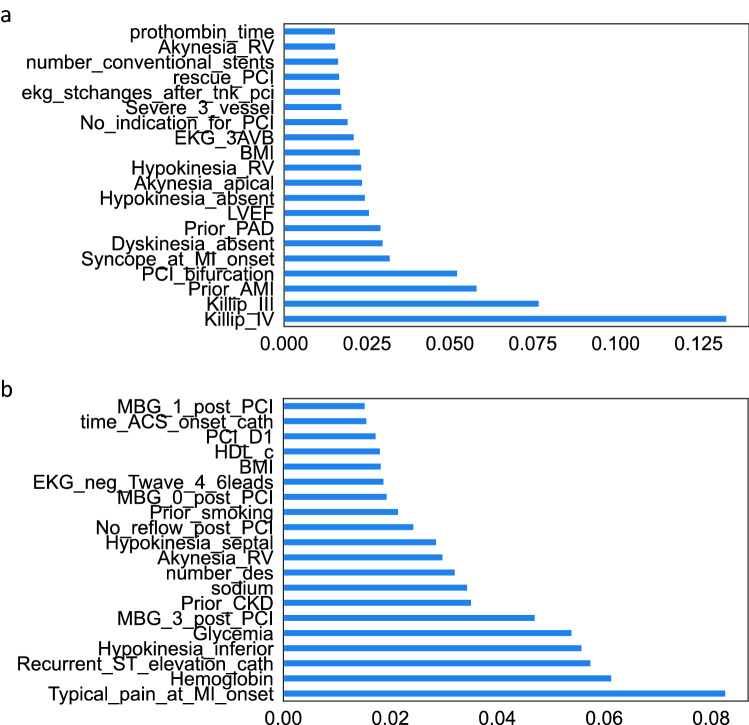
Predictive importance of key variables in *TabNet* model for *in-hospital* MACE. Legend: The most important predictors are listed in the y axis and explain at least 90% of variance in *TabNet* model for *in-hospital* MACE; the x axis represents the relative importance to the model. (**a**) the global cohort and (**b**) young individuals with ACS (≤ 55 years old). Prothrombin_time: prothrombin time; Akynesia_RV: right ventricular akinesia; number_conventional_stents: number of conventional stents; rescue_PCI: treatment with recue PCI due to absent reperfusion sygnals after thrombolysis in STEMI; PCI: percutaneous coronary interventions; severe_3_vessels: 3-vessel disease; no_indication_for_PCI: absent coronary artery lesions eligible for PCI; EKG_3AVB: 3rd degree AV block; BMI: body mass index; Hypokinesia_RV: right ventricular hypokinesia; Akinesia_apical: left ventricular apex akinesia; Hypokinesia_absent: no left ventricular wall showing hypokinesia; LVEF: left ventricular ejection fraction; PAD: peripheral artery disease; Dyskinesia_absent: no left ventricular wall showing dyskinesia; PCI_bifurcation: PCI in coronary forked segment; prior_AMI: past medical history of acute myocardial infarction; Killip_III: Killip class III; Killip_IV: Killip class IV; MBG_1_post_PCI: myocardial blush grade after PCI = 1; time_ACS_onset_cath: time between ACS symptoms onset and catheterization; PCI_D1: PCI of diagonal coronary artery; HDL_c: high density lipoprotein cholesterol; BMI: body mass index; EKG_neg_Twave_4_6leads: negative T waves in 4 to 6 leads; MBG_0_post_PCI: myocardial blush grade after PCI = 0; prior_smoking: past medical history of smoking; No_reflow_post_PCI: no-reflow phenomena observed after PCI; Hypokinesia_septal: left ventricular septal wall hypokinesia; Akynesia_RV: right ventricular akinesia; number_des: number of drug-eluting stents implanted during index PCI; sodium: blood sodium levels at admission; MBG_3_post_PCI: myocardial blush grade after PCI = 3; Glycemia: blood glucose levels at admission; Hypokinesia_inferior: left ventricular inferior wall hypokinesia; Recurrent_ST_elevation_cath: recurrent ST segment elevation during catetherization; Hemoglobin: blood hemoglobin levels at admission; Typical_pain_at_MI_onset: presence of typical pain pattern at the onset of myocardial infarction.

As shown in Fig. 1, 28 variables are included in the *TabNet* algorithm for the global cohort, and 20 are responsible for 91% of the model variance. In the yACS cohort, 24 variables were recruited, and 20 were responsible for 93% of the model variance. Among the top predictor variables that explain at least 90% of the model variance, Fig. 2 shows very different patterns for the *TabNet* algorithm trained in yACS subjects and *TabNet* trained in the global cohort. Risk models share three variables in common (blood glycemia, BMI and right ventricular akinesia), and the algorithm trained in yACS contains characteristics related to microvascular thrombosis and intraprocedural complications of catheterization.

### Long-term MACE with competing risks

Here, the clinical question is whether LTW_m_ would be better suited for *global follow-up* (observation window of the first 48 h) or whether it would perform better for *postdischarge* (from index ACS) risk prediction (observation window including *in-hospital* stay). *Postdischarge* models had 47 noncardiovascular deaths and 454 MACEs, while the *global follow-up* models had 92 noncardiovascular deaths and 631 MACEs among individuals with yACS.

**Figure 3:**
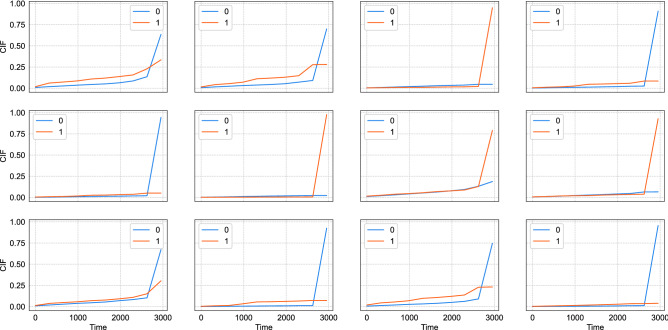
Estimated cumulative incidence functions (CIFs) for 12 random individuals with premature acute coronary syndrome (yACS) by using the *DeepHit* algorithm in *the postdischarge* horizon. Legend: zero (0) denotes CIF for noncardiovascular death, while one (1) means CIF for major cardiovascular adverse events (MACEs, cardiovascular deaths and recurrent ischemic events) occurring after discharge from index ACS hospitalization.

In *the postdischarge* models (available in Table 3), *CS-Cox* and *Fine-Gray* yielded the lowest C^*td*^ indexes in the test set, 0.602 (95% CI 0.556–0.649) and 0.612 (95% CI 0.564–0.663), while *DMGP* and *DeepHit* reached 0.685 (95% CI 0.639–0.725) and 0.722 (95% CI 0.678–0.760), respectively. *Global follow-up* models (Table 3) produced generally lower concordance indexes in the test set, 0.597 (95% CI 0.552–0.643), 0.601 (95% CI 0.559–0.660), 0.687 (95% CI 0.640–0.728) and 0.681 (95% CI 0.654–0.703) for *CS-Cox*, *Fine-Gray*, *DMGP* and *DeepHit*, respectively. *DeepHit* in *the postdischarge* horizon yielded the highest C^*td*^ index and the lowest IBS (0.0579), suggesting the highest accuracy. The CIFs of 12 random yACS individuals are depicted in Fig. 3. C^*td*^-indexes for the global cohort were similar to yACS both in *postdischarge* and *global follow-up* horizons.

**Table 3:** Time-dependent C-statistics (C^*td*^-index) for predicting long-term noncardiovascular deaths or MACEs (cardiovascular deaths and recurrent ischemic events) with competing risks occurring (i) after discharge in 2161 individuals with premature ACS (55 years old or younger), total number of *postdischarge* events: 47 noncardiovascular deaths and 454 MACEs; (ii) 48 h after index ACS hospital admission (*global follow-up* models), total number of events: 92 noncardiovascular deaths and 631 MACEs.

	C^*td*^-index (95% confidence interval)
***Post-discharge* horizon**	
*CS-Cox*	0.602 (95% CI 0.556–0.649)
*Fine-Gray*	0.612 (95% CI 0.564–0.663)
*DMGP*	0.685 (95% CI 0.639–0.725)
*DeepHit*	0.722 (95% CI 0.678–0.760)
***Global follow-up* horizon**	
*CS-Cox*	0.597 (95% CI 0.552–0.643)
*Fine-Gray*	0.601 (95% CI 0.559–0.660)
*DMGP*	0.687 (95% CI 0.640–0.728)
*DeepHit*	0.681 (95% CI 0.654–0.703)

Among the algorithms, only *CS-Cox* is easily interpretable; therefore, it was used to acquire a glance at the risk components for long-term MACE. As seen in Supplementary Tables S3 and S4, most short- and long-term MACE predictors differ significantly. Only the Killip class and prior CKD stood as predictors in both long-term *CS-Cox* and short-term *sLR*. The *CS-Cox* model in yACS individuals showed that drugs prescribed at discharge from index ACS, such as anticoagulants, furosemide and ticagrelor/prasugrel, are independently associated with MACEs. The atherosclerotic burden (Synthax score) and low ejection fraction were also linked to MACEs, but in yACS individuals, STEMI in index ACS showed reduced long-term risk compared to NSTEMI, and CABG as a treatment of index ACS was also associated with lower risk compared to PCI. Finally, the occurrence of non-fatal MACE during index ACS hospitalization was associated with an increased risk of long-term MACE. We did not observe differences in risk components for the global cohort and yACS individuals in the *CS-Cox* model.

### Combined models

In order to compare one-step models (full length follow-up) and two-step models (combination of short- and long-term predictive models), it was necessary to develop a mechanism to estimate the combined accuracy to predict MACE in the whole follow-up using the best short-term model and the best long-term model. Although C^td^-index and C-statistics are not comparable metrics, we generated a weighted score considering the number of events observed in the short-term (to weight the model with the best C-statistic) and the number of events observed in the long-term (to weight the model with the best C^td^-index). Combining *TabNet* algorithm in in-hospital horizon and *DeepHit* in *the postdischarge* horizon, this compound strategy led to the detection of 80% of events, compared to 69% in the *Global follow-up* using DMGP algorithm.

## Discussion

In this study, we found that individuals with yACS present different demographic characteristics and susceptibility to risk factors for MACEs compared to older subjects. We also identified that risk prediction models are optimized by using a compound strategy: (i) specific risk prediction rules for yACS individuals rather than targeted to the overall population; (ii) short-term predictions are highly efficient; and (iii) long-term prediction models should incorporate competing events and should be optimized by including *in-hospital* clinical data in the observation window. Roughly, the best model using this compound strategy led to the detection of 80% of events, compared to 69% by using general rules.

As mentioned, risk prediction rules are improved by the optimal selection of observation windows. This issue was recently reviewed by Lauritsen et al.^11^ and suggested that temporal *framing structures* are critical for successful risk prediction. In models for predicting sepsis, the authors suggested not only implementing optimal selection of observation/prediction windows but also including a sequential evaluation by using predictions made until the current timestep^17^. Indeed, Wong et al.^18^ suggested that a hospitalization-level risk score for sepsis based on the entire trajectory of predictions may enable more realistic evaluations. However, in clinical cardiology, risk scores are typically less dynamic and employ temporal *framing* suboptimally. By setting a short-term endpoint, we could identify important predictors of *in-hospital* MACE with a set of data gathered from the first two days of index ACS onset. In parallel, long-term risk prediction taking into account competing risks was optimized by including *predischarge* information, including prescription at discharge and *in-hospital* clinical events.

Another argument in favor of splitting two prediction windows is that we showed large differences between key predictors of MACE in the short term and the long term. In short-term models, the most important predictors of MACEs are symptoms at ACS presentation, microvascular thrombosis and intraprocedural complications of catheterization. Instead, in long-term models, the top predictors of worse clinical outcomes are mostly related to *in-hospital* outcomes, discharge medications, past medical history and severity of coronary artery lesions. In addition, splitting two prediction windows permits a flexible and dynamic way of dealing with clinical problems^17^.

It is important to mention that binary classification can provide predictions for a predetermined duration (e.g., *in-hospital* stay), useful for short-term outcomes where time to event is not an issue. Binary classification typically provides predictions for one predetermined duration, losing the interpretability and flexibility provided by modeling the event probabilities as a function of time. Hence, in clinical problems with a substantial amount of censoring, the use of survival models tends to be advantageous^19^. On the other hand, if censoring bias is not accounted for or the context can neutralize censoring, binary classification tends to maximize accuracy compared to survival models^11^. Therefore, the way to better fit a real-world scenario was to combine short-term classification with long-term survival.

For *in-hospital* MACE prediction, *TabNet* yielded the best results. The algorithm has been recently described and couples a deep neural network architecture and gradient descent-based optimization designed specifically for tabular data^16^. Together with the great predictive capacity, it also enables interpretability. Although no causality can be attributed to top predictors, they are consistent with the most prevalent risk factors for MACEs among yACS^4,20^. As seen by others^20^, we observed that variables of interest for predicting MACEs in individuals with premature ACS differed from the top predictors among the global cohort and older subjects. In addition, our findings for *in-hospital* MACE prediction suggest both a strategy to predict events in a specific subset and a better predictive model. To exemplify this quote, the GRACE score was slightly better among older subjects compared to yACS to predict the risk of *in-hospital* MACE with AUC of 0.829 (95% CI 0.792–0.867) (data not shown), but still lower than *Tabnet* algorithm. As the GRACE score was designed to predict in-hospital death or cardiovascular events at 6 months, its performance in long-term risk prediction (> 2-year follow-up) is very limited^5^.

Among the long-term models, *DeepHit* was the most accurate. *DeepHit* is a multitask network that makes no linear assumptions during the predictive process, allowing for the possibility that the relationship between covariates and risks changes over time^14^. Although such architecture improves predictive ability and flexibility to deal with competing risks compared to *CS-Cox* and *Fine-Gray* models, it is not possible to interpret which variables are recruited at each step. However, among the long-term predictors of MACEs using *CS-Cox,* we identified that yACS may be at higher risk when prescribed at discharge drugs such as ticagrelor or prasugrel than clopidogrel. These observations contradict the findings from major clinical trials such as PLATO^21^ and TRITON-TIMI-38^22^ but should be explored in other real-world scenarios with appropriate techniques for neutralizing any potential selection bias.

There are limitations in this study that should be acknowledged. First, the observational and retrospective design of this study limits any potential causal conclusions. Second, the definition of yACS is not consensus; while some consider an age threshold of 55 years old, others consider 50 or 45 years old^4,6,23^. Third, guideline-specific medication and ongoing management of risk factors are of unquestionable importance. Unfortunately, data on medical therapy beyond discharge was not available and this represents an important limitation. Forth, our models were trained in a relatively small cohort. Although the B-CaRe:QCO yACS cohort is among the largest cohorts of yACS, some algorithms, such as *DeepHit*, *DMGP*, and *TabNet,* were originally developed in datasets of > 10,000 individuals^14,16,24^. Our results suggest that these algorithms also perform well in smaller datasets, and we did our best to maximize external validity by using cross-validation and resampling techniques. The main advantage of our cohort is that we systematically included all subjects admitted due to ACS in public hospitals from Brasília (Brazil).

In summary, we found that individuals with premature ACS share considerable morbidity and show unique epidemiologic features compared to those of older subjects. In this study, we also identified that risk prediction models are optimized by using specific risk prediction rules for yACS individuals in two windows: a short-term window and a long-term window that incorporate competing events and *in-hospital* clinical data within the observation window. It is critical to better understand risk factors within this subgroup to allow public health initiatives that mitigate the economic burden aroused by yACS^4,6^. Risk prediction-enhanced clinical care could turn into a framework for intensified clinical surveillance in individuals predicted to be high risk^5^.

## Methods

### Study design and participants

The set of individuals was selected from the *B-CaRe:QCO* (*Brasilia Cardiovascular Registry for Quality of Care and Outcomes*), a retrospective registry of 6341 subjects with ACS (n = 2242 with yACS). The *B-CaRe:QCO* study included consecutive individuals admitted to public hospitals in Brasília (DF) with ACS who underwent coronarography up to 48 h after hospital admission from January 2011 to February 2020. At that time, all coronarographies were carried out in *Hospital de Base* (Brasília-DF, Brazil) and *Instituto de Cardiologia* (Brasília-DF, Brazil). We excluded 17 individuals who died within the first 48 h.

Enrolled subjects experienced therapies based on guidelines for the treatment of ACS^25^. Attending physicians made all therapeutic decisions and were blinded to the study evaluations. Most individuals admitted due to STEMI (n = 1659 with premature ST-elevation myocardial infarction [STEMI]) were treated by primary percutaneous coronary intervention (pPCI) or pharmacoinvasive strategy.

The methods were performed in accordance with relevant guidelines and regulations, and approved by the Institutional Ethics Review Board from *Instituto de Gestão Estratégica em Saúde do Distrito Federal* (IGESDF) (study protocol approval number [CAAE] 28530919.0.1001.8153).

For predicting *in-hospital* MACE (defined as cardiovascular deaths or recurrent ACS) occurring 48 h after hospital admission, the observation window comprised the first 48 h after hospital admission. The yACS dataset was divided into a training/validation set (70%, n = 1569) and a test set (30%, n = 673). Short-term models (STW_m_) were trained and validated in a fivefold cross-validation framework with upsampling to mitigate outcome imbalance, a setting that usually produces classifier’s bias towards the majority class^26,27^. STW_m_ was then evaluated in the test set.

To predict long-term outcomes with competing risks (noncardiovascular deaths vs MACE), two contexts were evaluated: (i) *postdischarge*, where an observation window included the whole period of index hospitalization (mean of 5 ± 2 days) and the outcomes were observed from hospital discharge to the end of follow-up (median of 6.67 years); (ii) *global follow-up*, where the observation window included only the first 48 h and the outcomes observation period began at 48 h and extended to the end of follow-up. A training/validation set (n = 1513) and test set (n = 648) included individuals alive at discharge and were used to train and validate long-term window models (LTW_m_). LTW_m_ was repeated over five cross-validation folds and then assessed in the test set.

To better understand model accuracy and differences in key predictors for short-term MACE between the yACS and older subjects, we also created models using the *global cohort* (n = 6341) by splitting a training/validation set (n = 4439) and a test set including only 673 individuals in the yACS test set (remaining 1229 individuals older than 55 years were not included in the test set to prevent sampling imbalance). Again, we used fivefold cross-validation with upsampling for STW_m_ and evaluated the model in the yACS test set (n = 673).

### Clinical definitions and outcome assessment

Current smokers were defined as those who had smoked at least 100 cigarettes during their lifetime and were smoking at least one year before ACS onset, according to the National Health Interview Survey (NHIS) definition^28^. Ex-smoking status was defined as smoking cessation for at least the last 6 months. Diabetes was defined as the use of antidiabetic medications, prior diagnosis of diabetes, or glycosylated hemoglobin (HbA1c) ≥ 6.5% at hospital admission. Patients were considered hypertensive if they were taking any antihypertensive medication or presented systolic blood pressure (SBP) ≥ 140 mm Hg or diastolic blood pressure (DBP) ≥ 90 mmHg. The anthropometric measurements obtained were body weight (kg), height (m), and waist circumference (cm). The Killip class and GRACE scores for *in-hospital* MACEs were evaluated in all enrolled patients^29^.

Clinical outcomes were assessed by checking electronic health records (EHRs). Information about the cause of death and clinical events was obtained from the death certificate or medical records. The following adverse cardiac events for both STW_m_ and LTW_m_ were considered: cardiovascular deaths and recurrent ACS (MACE). For STW_m_, those who had any event during follow-up were marked as 1, and those who did not were coded as 0. For LTW_m,_ we considered a competing event approach in survival analyses, i.e., individuals were followed until their deaths, the occurrence of recurrent ischemic events or the end of follow-up (last visit to the outpatient clinic registered in EHRs). Reinfarction was defined as the occurrence of new ischemic symptoms during the first 28 days after index MI associated with a > 20% increase in cTn levels after a 3-to-6-h interval from symptoms^30^.

### Models and variable selection

A domain-knowledge-driven approach was first used to select variables. From 186 variables at baseline, we excluded variables with no potential causal link with the outcomes and included those proven as predictors in previous models, leaving the remaining 108 variables. Variables were included only if they were unambiguous in their interpretation and recorded in a structured (numeric/binary) format.

After this, a data-driven approach took place and consisted of an automated process based on actual data and the relevance of each variable to a specific outcome^31^. For most of the STW_m_ and LTW_m_, we used a fully automated process incorporated into the algorithms. When selection could not be performed automatically, we followed guidelines as proposed by Belsley et al.^32^: in the case of high correlation between variables (partial R^2^ ≥ 0.5 in univariate regression with MACE[= 1] as the dependent variable or variance inflation factor [VIF] > 10), we dropped the variables with lower R^2^. Information-gain ranking was used to evaluate the worth of each variable by measuring the entropy gain with respect to the outcome, followed by ranking the attributes by their individual evaluations. Considering the *tradeoffs* between the cost of information and information gain, only attributes resulting in information gain higher than 0.01 were subsequently used in STW_m_ and LTW_m_. Variable selection was performed in the training/validation dataset.

Missing values (MVs) were relatively rare (2.7% of B-CaRe:QCO data). We handled MVs with multiple imputations directly in the training/validation dataset by using boosted trees. Real life datasets are likely to have horizontal data segments where records have higher similarity and attribute correlations than the similarity and correlations of the whole data set. Boosted trees can explore these segments and improves the imputation accuracy by taking a global approach in the sense that it imputes missing values using the whole dataset, instead of a horizontal segment of it, unlike the family of k-NN imputation techniques^33,34^. Only a few variables showed MV frequencies ≥ 10% (plasma TSH, free T4 and urea). Imputation using boosted trees fills each column by treating it as a regression problem. We did not impute missing values for the outcomes.

### Predictive algorithms

For predicting short-term outcomes, we used *XGBoost*^35^, *random forests*^36^, and *TabNet*^16^. *Random forests*, based on decision trees, rank variable importance on the selection frequency of the variable as a decision node and generally show good performance for classification problems in tabular data with a single outcome^5^. *XGBoost* is also based on decision trees and uses gradient descent-based optimization^35^. *TabNet* has an interpretable canonical deep tabular data learning architecture, merging both deep learning and gradient descent-based optimization. The observation window was considered the first 2 days upon hospital admission and encompassed past medical history, emergency room data and coronarography. We compared models with the benchmark GRACE score^37^, recalibrated using regression coefficients of risk factors derived from logistic regressions (LR) as described elsewhere^5^ (details in below).

For long-term outcomes, we used the following survival algorithms with competing risks: cause-specific Cox-proportional hazards model (*CS-Cox*)^38^, Fine-Gray proportional subdistribution hazards model (*Fine-Gray*)^39^, deep multitask Gaussian process (*DMGP*)^24^, and *DeepHit*^14^. *CS-Cox* and *Fine-Gray* assume linear proportional hazards, *DMGP* assumes the underlying stochastic process to follow the Gaussian process, and *DeepHit* employs a network architecture that makes no assumptions about the relationship between predictors and outcomes.

Each model’s hyperparameters were determined using the grid search method^40^ and fivefold cross-validation for STW_m_ and LTW_m_. STW_m_ were generated with upsampling to mitigate outcome imbalance. Performance in the validation set is reported as the mean of 5-folds. A full description of variable selection, hyperparameters and model architectures can be found below.

### Model development process

To develop the prognostic models, B-CaRe:QCO data were extracted into a labelled dataset containing the independent variables (using the patients’ clinical records at their baseline dates or during index hospitalization) and all dependent variables (occurrence of a composite endpoint of death due to cardiovascular causes and recurrent ACS following the baseline date).

We implemented a grid search for the hyperparameter optimization using the method reported by Bergstra and Bengio^40^. This requires the operator to specify a range of values for each hyperparameter, and all possible combinations of the hyperparameters are investigated, with the combination corresponding to the highest cross-validation performance metric (in this case, maximization of the *C-statistics* being chosen for the final model). The justification for selecting the hyperparameters that maximise the *C-statistics* is that this is less affected when the labelled data are unbalanced compared to using accuracy as a metric. When the classes are unbalanced, it is also a common strategy to oversample the rare label data and undersample the common label data, as many machine learning models can be sensitive to unbalanced data^41^. Below, we describe in further detail the algorithms used.

### Short-term predictive algorithms for classification

*Random forests*. For the hyperparameter grid search, we investigated ntree = 50, 150, and 350; mtry from 5 up to the maximum number of variables in increments of 5; max depth = 2, 4, 6, 8, and 10; and row samples of 90%, 95% and 100%. The chosen (optimal) random forest model had the following hyperparameters: ntree = 350, mtry = 25, max depth = 5 (up to 5 variable interactions were used by the model) and row sample fraction of 0.95 (95% of the data points were used to train each tree).

*XGboost*. The grid search for the hyperparameters investigated in our models were ntree = 25, 50, 75 and 100; max depth = 2, 3, 4, 6 and 8; and the minimum observations per node was 5, 10, 20, and 40. The gradient boosting machine model was chosen to have a Bernoulli distribution, and the chosen model had the following hyperparameters: ntree = 50, max depth = 3 (up to 3 variable interactions were used by the model), and the minimum number of observations per node was 10. *XGBoost* was implemented in Python.

*TabNet*. We used a canonical deep neural network (DNN) architecture for tabular data described by Arik et al.^16^. Briefly, *TabNet* is trained using gradient descent-based optimization and uses sequential attention to choose which features to reason from at each decision step, enabling (i) interpretability, (ii) more accurate and faster learning and (iii) flexible integration into end-to-end learning. Through sparse and instancewise selection (*sparsemax* is used for normalization of the coefficients) of features with the highest impact on outcomes, the learning capacity of a decision step is not wasted on irrelevant ones, and thus the model becomes more parameter efficient. *TabNet* also constructs a sequential multistep architecture, where each step contributes to a portion of the decision based on the selected features, improves the learning capacity via nonlinear processing of the selected features, and mimics ensembling via higher dimensions. The *TabNet* encoder is composed of a feature transformer, an attentive transformer and feature masking. A split block divides the processed representation to be used by the attentive transformer of the subsequent step as well as for the overall output. For each step, the feature selection mask provides interpretable information about the model’s functionality, and the masks can be aggregated to obtain global feature important attributions. The *TabNet* decoder is composed of a feature transformer block at each step. Each feature transformer block is composed of a 4-layer network, where 2 are shared across all decision steps and 2 are decision step-dependent. Each layer is composed of a fully connected (FC) layer, ghost batch normalization (BN) and gated linear unit (GLU) nonlinearity. We used standard classification (*softmax* cross entropy) loss functions, and we trained the model until convergence using unsupervised pretraining. The final *TabNet* model was implemented in *a PyTorch* environment and had the following configuration: *Adam* optimizer with a learning rate of 0.02 and a decay rate of 0.9 every 10 interactions, *Glorot* uniform initialization, batch size of 256, Max epoch 1000, workers at zero, momentum of 0.9, N_*steps*_ = 8, γ = 2.0, and weight at 1 (automated sampling).

*Logistic regression models*. We built a series of stepwise logistic regression models to predict *in-hospital* MACEs.

### Long-term predictive models – survival with competing risks

*Cause-specific Cox-proportional hazards model (Cox) and Fine-Gray proportional subdistribution hazards model (Fine-Gray)*. The Cox model relates the covariates to the hazard function of the outcome of interest and not directly to the survival times themselves. The covariates have a relative effect on the hazard function because of the use of the logarithmic transformation, and the regression coefficients are interpreted as log-hazard ratios. The hazard ratio is equal to the exponential of the associated regression coefficient^38^. Competing risks imply that a subject can experience one of a set of different events or outcomes. In this case, two different types of hazard functions are of interest: the cause-specific hazard function and the subdistribution hazard function. The cause-specific hazard function indicates the instantaneous rate of occurrence of the *k*th event in subjects who are currently event free (i.e., in subjects who have not yet experienced any of the different types of events). Considering two types of events, death attributable to cardiovascular causes and death attributable to noncardiovascular causes, the cause-specific hazard of cardiovascular death denotes the instantaneous rate of cardiovascular death in subjects who are still alive. It denotes the instantaneous risk of failure from the *k*th event in subjects who have not yet experienced an event of type *k*. There is a distinct cause-specific hazard function for each of the distinct types of events and a distinct subdistribution hazard function for each of the distinct types of events. In settings in which competing risks are present, two different hazard regression models are available: modeling the cause-specific hazard and modeling the subdistribution hazard function. The second model has also been described as a *cumulative incidence function* (CIF) regression model, which means that the subdistribution hazard model allows one to estimate the effect of covariates on the cumulative incidence function for the event of interest. However, it is recommended to use the Fine-Gray (FG) subdistribution hazard model when the focus is on estimating incidence or predicting prognosis in the presence of competing risks, since this model generally shows better accuracy than the *Cox* model. The *(cause-specific) cumulative incidence function* (CIF) expresses the probability that a particular event k^*^ occurs on or before time t^*^ conditional on covariates x*. Since *true* CIF is not known, the model utilizes *estimated* CIF to compare the risk of events occurring and to assess how models discriminate across cause-specific risks among patients. Model performance was calculated by using the time-dependent concordance index C^*td*^^42^ (C^*td*^-index). *Cox* and *FG* benchmarks were run using the R libraries *survival* and *cmprsk*. We estimated the time-dependent *C*^*td*^* index* for the survival analysis methods under consideration using the function *cindex* of the R package *pec*.

*A deep multitask Gaussian process (DMGP)*^24^ is a nonparametric Bayesian model for survival analysis that relies on a conception of the competing risks problem as a multitask learning problem; i.e., it models the cause-specific survival times as the outputs of a random vector-valued function, the inputs to which are the patients’ covariates. This allows the model to learn a “shared representation” of survival times with respect to multiple related comorbidities. Inference of patient-specific posterior survival distribution is conducted via a variational Bayes algorithm. By using *inducing variables* to derive a variational lower bound on the marginal likelihood of the observed time-to-event data, which is maximized using the adaptive moment estimation algorithm (*Adam*). Hyperparameters Θ_Z_ and Θ_T_ were tuned using the *offline* B-CaRe:QCO dataset, and for any out-of-sample patient with all covariates, *DMGP* evaluates posterior probability density by direct Monte Carlo sampling. Hyperparameters were calibrated by maximizing the marginal likelihood of posterior probability density. *DMGP* was implemented in Python.

*DeepHit* trains a neural network to learn the estimated joint distribution of survival time and event while capturing the right-censored nature inherent in survival data^14^. The network is trained by using a loss function that exploits both survival times and relative risks. *DeepHit* makes no assumptions about the underlying stochastic process and allows for the possibility that the relationship between covariates and risks changes over time. *DeepHit* is a multitask network that consists of a shared subnetwork and *K* cause-specific subnetworks, differing from that of a conventional multitask network in two ways: (i) it utilizes a single *softmax* layer as the output layer of *DeepHit* to ensure that the network learns the joint distribution of *K* competing events, not the marginal distributions of each event; (ii) it keeps a residual connection from the input covariates into the input of each cause-specific subnetwork. To train *DeepHit*, a total loss function *L*_*Total*_ is specifically designed to handle censored data. This loss function is the sum of two *terms L*_*Total*_ = *L*_1_ + *L*_2_; *L*_1_ is the log-likelihood of the joint distribution of the first hitting time and event; *L*_2_ incorporates a combination of cause-specific ranking loss functions that adapts the idea of concordance. The hyperparameters for *L*_*Total*_ were selected based on the discriminative performance on the validation set. Early stopping was performed based on the total loss. *DeepHit* is a 4-layer network consisting of 1 fully connected layer for the shared subnetwork and 2 fully connected layers for each cause-specific subnetwork and a *softmax* layer as the output layer. For hidden layers, the number of nodes was set as 3, 5, and 3 times the covariate dimension for layers 1, 2, and 3, respectively, with the *ReLu* activation function. The network was trained by backpropagation via the *Adam* optimizer with a batch size of 50 and a learning rate of 0.0001. A dropout probability of 0.6 and Xavier initialization were applied for all layers. *DeepHit* was implemented in a *TensorFlow* environment in Python.

### Statistical analysis

STW_m_ were compared using *accuracy* and *C-statistics* for their performance on the test and validation datasets. We calculated the median performance and 95% confidence intervals (CIs) for the *C-statistics* for each algorithm. We built models with the training/validation set and finally evaluated the model on the test set to estimate performance. STW_m_ was compared to the *C-statistics* obtained by the recalibrated GRACE score^37^. LTW_m_ evaluates each individual’s cumulative incidence function (CIF), also known as the *subdistribution function*. CIF is commonly used in settings with competing risks and refers to the probability of a particular event during follow-up. CIFs are used to evaluate the case-specific concordance, and this concept is used to derive a performance metric to compare LTW_m_, the time-dependent concordance index C^*td*^^42^. The C^*td*^-index measures the extent to which the ordering of actual survival times of pairs agrees with the ordering of their predicted risk (further information is available in Supplemental Methods). A confidence interval for the C^*td*^ index is derived using the jackknife method on correlated one-sample U-statistics. Jackknife method was used because it is less computationally expensive than bootstrapping. The integrated Brier score (IBS) was also used as an LTW_m_ evaluation measure. Normally distributed data are presented as the mean ± SD, and skewed data are presented as the median [interquartile range (IQR)]. Normality of distribution and variances were checked using histograms, Kolmogorov-Smirnoff test, normal probability plots and residual scatter plots. Chi-square or two-tailed *t*-tests were used for comparison of baseline data. *P* values < 0.05 were considered significant. Analyses were carried out using R[v4.0.1] and Python[v3.8], and the packages used are described in the Supplemental Methods.

### IRB approval and patient consent

The study proceedings are in accordance with the Helsinki Declaration and the study was approved by the Institutional Ethics Review Board (IRB) from *Instituto de Gestão Estratégica do Distrito Federal* (IGESDF) (study protocol approval number [CAAE] 28530919.0.1001.8153). Since this is a retrospective study, the IRB approved the waiver of participants informed consent as long as data is captured anonymously.

## Supplementary Information

Supplementary Information.

## Data Availability

Codes are available at https://github.com/lsergiocarvalho/openwindowACS. All requests for raw and analyzed data and related materials, excluding programming codes, will be reviewed by the Clarity Healthcare Intelligence legal department to verify whether the request is subject to any intellectual property or confidentiality obligations. Requests for patient-related data can be considered upon request (contact: contato@clarityhealth.com.br). Any data and materials that can be shared will be released via a Material Transfer Agreement.

## References

[1] Arora S, Stouffer GA, Kucharska-Newton AM (2019). Twenty year trends and sex differences in young adults hospitalized with acute myocardial infarction. Circulation.

[2] Gupta A, Wang Y, Spertus JA (2014). Trends in acute myocardial infarction in young patients and differences by sex and race, 2001 to 2010. J. Am. Coll. Cardiol..

[3] Zeitouni M, Clare RM, Chiswell K (2020). Risk factor burden and long-term prognosis of patients with premature coronary artery disease. J. Am. Heart Assoc..

[4] Dreyer RP, Raparelli V, Tsang SW (2021). Development and validation of a risk prediction model for 1-year readmission among young adults hospitalized for acute myocardial infarction. J. Am. Heart Assoc..

[5] de Carvalho LSF, Gioppato S, Fernandez MD (2020). Machine learning improves the identification of individuals with higher morbidity and avoidable health costs after acute coronary syndromes. Value Health.

[6] Sagris, M., Antonopoulos, A. S., Theofilis, P. *et al.* Risk factors profile of young and older patients with Myocardial Infarction. *Cardiovasc. Res.* (2021). 10.1093/cvr/cvab264 34358302

[7] Yanase T, Sakakura K, Taniguchi Y (2021). Comparison of clinical characteristics of acute myocardial infarction between young (< 55 Years) and older (55 to < 70 Years) patients. Int. Heart J..

[8] Alexim GA, Rocha LF, Dobri GP (2022). Clinical and economic impact of coronary artery bypass graft and percutaneous coronary intervention in young individuals with acute coronary syndromes and multivessel disease: A real-world comparison in a middle-income country. Front. Cardiovasc. Med..

[9] Oellgaard J, Gaede P, Rossing P (2018). Reduced risk of heart failure with intensified multifactorial intervention in individuals with type 2 diabetes and microalbuminuria: 21 years of follow-up in the randomised Steno-2 study. Diabetologia.

[10] Gaede P, Lund-Andersen H, Parving HH, Pedersen O (2008). Effect of a multifactorial intervention on mortality in type 2 diabetes. N. Engl. J. Med..

[11] Lauritsen SM, Thiesson B, Jorgensen MJ (2021). The Framing of machine learning risk prediction models illustrated by evaluation of sepsis in general wards. NPJ Digit. Med..

[12] Kuo RN, Dong YH, Liu JP, Chang CH, Shau WY, Lai MS (2011). Predicting healthcare utilization using a pharmacy-based metric with the WHO’s Anatomic Therapeutic Chemical algorithm. Med. Care.

[13] Lauffenburger JC, Mahesri M, Choudhry NK (2020). Use of data-driven methods to predict long-term patterns of health care spending for medicare patients. JAMA Netw. Open..

[14] Lee, C., Zame, W. R., Yoon, J. & Van Der Schaar M. DeepHit: A deep learning approach to survival analysis with competing risks. In *Paper presented at: XXXII Association for the Advancement of Artificial Intelligence (AAAI) Conference* (2018).

[15] McCaw, Z. R., Claggett, B. L., Tian, L. *et al.* Practical recommendations on quantifying and interpreting treatment effects in the presence of terminal competing risks: A review. *JAMA Cardiol.* (2021). 10.1001/jamacardio.2021.4932 34851356

[16] Arik, S. O. & Pfister, T. TabNet: Attentive interpretable tabular learning. Association for the Advancement of Artificial Intelligence (2020).

[17] Lauritsen SM, Kalor ME, Kongsgaard EL (2020). Early detection of sepsis utilizing deep learning on electronic health record event sequences. Artif. Intell. Med..

[18] Wong A, Otles E, Donnelly JP (2021). External validation of a widely implemented proprietary sepsis prediction model in hospitalized patients. JAMA Intern. Med..

[19] Kvamme H, Borgan Ø, Scheel I (2019). Time-to-event prediction with neural networks and cox regression. J. Mach. Learn. Res..

[20] Lei L, Bin Z (2019). Risk factor differences in acute myocardial infarction between young and older people: A systematic review and meta-analysis. Int. J. Cardiovasc. Sci..

[21] Wallentin L, Becker RC, Budaj A (2009). Ticagrelor versus clopidogrel in patients with acute coronary syndromes. N. Engl. J. Med..

[22] Wiviott SD, Braunwald E, McCabe CH (2007). Prasugrel versus clopidogrel in patients with acute coronary syndromes. N. Engl. J. Med..

[23] Divakaran S, Singh A, Biery D (2020). Diabetes is associated with worse long-term outcomes in young adults after myocardial infarction: The partners YOUNG-MI registry. Diabetes Care.

[24] Alaa, A. M. & van der Schaar, M. Deep multi-task gaussian processes for survival analysis with competing risks. In *30th Conference on Neural Information Processing Systems* (2017).

[25] Ibanez B, James S, Agewall S (2018). 2017 ESC Guidelines for the management of acute myocardial infarction in patients presenting with ST-segment elevation: The Task Force for the management of acute myocardial infarction in patients presenting with ST-segment elevation of the European Society of Cardiology (ESC). Eur. Heart J..

[26] Dubey R, Zhou J, Wang Y, Thompson PM, Ye J (2014). Alzheimer's Disease Neuroimaging Initiative. Analysis of sampling techniques for imbalanced data: An n = 648 ADNI study. Neuroimage.

[27] Moniz N, Branco P, Torgo L (2017). Resampling strategies for imbalanced time series forecasting. Int. J. Data Sci. Anal..

[28] Ryan H, Trosclair A, Gfroerer J (2012). Adult current smoking: Differences in definitions and prevalence estimates–NHIS and NSDUH, 2008. J. Environ. Public Health.

[29] Fox KA, Dabbous OH, Goldberg RJ (2006). Prediction of risk of death and myocardial infarction in the six months after presentation with acute coronary syndrome: Prospective multinational observational study (GRACE). BMJ.

[30] Thygesen K, Alpert JS, Jaffe AS (2018). Fourth universal definition of myocardial infarction (2018). Circulation.

[31] Heinze G, Wallisch C, Dunkler D (2018). Variable selection—A review and recommendations for the practicing statistician. Biom. J..

[32] Belsley DA, Kuh E, Welsch RE (2013). Regression Diagnostics: Identifying Influential Data and Sources of Collinearity.

[33] Atiq R, Fariha F, Mahmud M, Yeamin SS, Rushee KI, Rahim S (2022). A comparison of missing value imputation techniques on coupon acceptance prediction. Int. J. Inf. Technol. Comput. Sci. (IJITCS).

[34] Rahman G, Islam Z (2013). Missing value imputation using decision trees and decision forests by splitting and merging records: Two novel techniques. Knowl. Based Syst..

[35] Chen, T. & Guestrin, C. XGBoost: A scalable tree boosting system. In *Paper presented at: KDD ‘16: Proceedings of the 22nd ACM SIGKDD International Conference on Knowledge Discovery and Data Mining, San Francisco* (2016).

[36] Breiman L (2001). Random forests. Mach. Learn..

[37] Granger CB, Goldberg RJ, Dabbous O (2003). Predictors of hospital mortality in the global registry of acute coronary events. Arch. Intern. Med..

[38] Austin PC, Lee DS, Fine JP (2016). Introduction to the analysis of survival data in the presence of competing risks. Circulation.

[39] Fine JP, Gray RJ (1999). A proportional hazards model for the subdistribution of a competing risk. J. Am. Stat. Assoc..

[40] Bergstra J, Bengio Y (2012). Random search for hyper-parameter optimization. J. Mach. Learn. Res..

[41] Krawczyk B (2016). Learning from imbalanced data: Open challenges and future directions. Prog. Artif. Intell..

[42] Antolini L, Boracchi P, Biganzoli E (2005). A time-dependent discrimination index for survival data. Stat. Med..

