# Supplementary material for: The framing of time-dependent machine learning models improves risk estimation among young individuals with acute coronary syndromes
Source: Sci Rep. 2023 Jan 19;13:1021. doi: 10.1038/s41598-023-27776-0 (PMC9852445; doi:10.1038/s41598-023-27776-0)
Supplement: Supplementary file 1 — Supplementary Information. [file 41598_2023_27776_MOESM1_ESM.docx]

**Supplemental Methods Section**

**The *Framing* of time-dependent machine learning risk prediction models among young individuals with acute coronary syndromes**

**Supplemental data:** 1 file, 5 pages, 4 tables

**Supplementary Table S1.** Incidence of death and recurrent cardiovascular events among individuals with premature ACS and older subjects

|  | **Number of events** | |  | **Per 1000 patients-years** | |
| --- | --- | --- | --- | --- | --- |
|  | **>55 y**  (n=4099) | ≤**55y**  (n=2242) | total | **>55 y**  (n=4099) | ≤**55y**  (n=2242) |
|  |  |  |  |  |  |
| *In-hospital* deaths | 233 | 81 | 314 | - | - |
| *In-hospital* CV deaths | 104 | 39 | 143 | - | - |
| *In-hospital* MIs | 389 | 141 | 530 | - | - |
| Post-discharge deaths | 395 | 89 | 484 | 15.06 | 5.97 |
| Post-discharge CV deaths | 110 | 42 | 152 | 4.46 | 3.02 |
| Post-discharge MIs | 771 | 412 | 1183 | 29.21 | 27.60 |
| Global deaths | 628 | 170 | 798 | 23.96 | 11.39 |
| Global CV deaths | 214 | 78 | 292 | 8.14 | 5.24 |
| Global non-CV deaths | 414 | 92 | 506 | 15.82 | 5.99 |
| Global MI during follow-up |  |  |  |  |  |
| STEMI | 525 | 132 | 657 | 20.04 | 8.84 |
| UA/NSTEMI | 635 | 421 | 1056 | 24.27 | 28.17 |
|  |  |  |  |  |  |

**Supplementary Table S2.** Top predictors to explain 90% of variance in logistic regression model for *in-hospital* MACE within global cohort

|  | Odds ratio | 95% CI | | p-value |
| --- | --- | --- | --- | --- |
|  |  | lower CI | upper CI |  |
|  |  |  |  |  |
| Syncope at ACS onset | 4,606 | 1,580 | 12,63 | 0,0038 |
| Prior ethylic habit | 0,502 | 0,253 | 0,938 | 0,0386 |
| Prior CKD | 2,080 | 1,218 | 3,508 | 0,0066 |
| Prior CABG | 3,011 | 1,095 | 8,565 | 0,0488 |
| EKG ST-segment elevation | 17,67 | 1,632 | 483 | 0,0383 |
| EKG Q wave v4r | 0,307 | 0,161 | 0,561 | 0,0002 |
| SBP at admission (each 1 mmHg) | 1,005 | 0,999 | 1,012 | 0,0844 |
| Killip class (each class) | 3,054 | 2,589 | 3,625 | <0,00001 |
| BMI | 1,027 | 0,999 | 1,065 | 0,0630 |
| Recurrent ST elevation during catheterization* | 71,63 | 24,06 | 227 | <0,00001 |
| MINOCA | 9,356 | 4,181 | 20,30 | <0,00001 |
| MBG=3 (post-PCI) | 0,763 | 0,668 | 0,871 | 0,0001 |
| Stent number during PCI (each additional stent) | 1,260 | 1,086 | 1,456 | 0,0020 |
| Apex hypokinesia | 2,246 | 1,184 | 4,119 | 0,0107 |
| Presence of dyskinesia (any wall) | 3,154 | 2.028 | 4.902 | <0,00001 |
| Troponin peak (per 1000 mUI/L) | 1,033 | 1,017 | 1,048 | <0,00001 |
| Blood glycemia (per 10 mg/dL) | 1,301 | 1,098 | 1,503 | 0,0030 |
|  |  |  |  |  |

* Individuals with UA/NSTEMI did not present ST elevation during catheterization. Twenty-seven STEMI subjects receiving thrombolysis had recurrent ST-elevation during catheterization (not including recue PCI)

**Supplementary Table S3.** Top predictors to explain at least 90% of variance in logistic regression model for *in-hospital* MACE within individuals with premature ACS (≤ 55 years-old)

|  | Odds ratio | 95% CI | | p-value |
| --- | --- | --- | --- | --- |
|  |  | lower CI | upper CI |  |
|  |  |  |  |  |
| Syncope at ACS onset | 6,310 | 1,075 | 35,51 | 0.0051 |
| Prior CKD | 3,262 | 1.286 | 10,76 | 0.0062 |
| Killip class (each class) | 6,931 | 3,116 | 15,31 | <0.0001 |
| Increasing duration of catheterization (each 10 min) | 1,412 | 1,040 | 1,890 | 0.0181 |
| Recurrent ST elevation during catheterization* | 246 | 40,55 | 2145 | <0.0001 |
| Late catheterization** | 3,236 | 1,088 | 8,631 | 0.0246 |
| MBG=3 (post PCI) | 0.496 | 0.239 | 0,998 | 0.0482 |
| Presence of dyskinesia (any wall) | 5.435 | 1.779 | 22.72 | 0.0076 |
| Blood glycemia at admission (per 10 mg/dL) | 2,104 | 1,378 | 3,232 | 0.0006 |
|  |  |  |  |  |

* Any individual with UA/NSTEMI did present ST elevation during catheterization. Eleven STEMI subjects receiving thrombolysis had recurrent ST-elevation during catheterization (not including recue PCI)

** Late catheterization defined as: after 12h after symptoms onset for STEMI; and after 24h after symptoms onset for UA/NSTEMI.

ACS: acute coronary syndromes; MBG: myocardial blush grade; PCI: percutaneous coronary intervention

**Supplementary Table S4.** Top predictors to explain at least 50% of variance in *cause-specific* *Cox* regression model for *post-discharge* MACE within individuals with premature ACS (≤ 55 years-old)

|  |  | 95% CI | |  |
| --- | --- | --- | --- | --- |
|  | HR | Lower bound | Upper bound | p |
| Age | 1.0065 | 1.0007 | 1.0192 | 0.0350 |
| Prior T2DM | 1.1132 | 0.9515 | 1.3024 | 0.1806 |
| Prior CKD | 1.3338 | 0.9719 | 1.8305 | 0.0744 |
| Prior PCI/stenting | 1.7116 | 1.3570 | 2.1589 | <0.0001 |
| Prior CABG | 1.3141 | 0.9627 | 1.7937 | 0.0853 |
| (Prasugrel or Ticagrelor) vs clopidogrel (at discharge) | 1.1795 | 1.0321 | 1.3996 | 0.0176 |
| Anticoagulation (at discharge) | 2.2196 | 1.6742 | 2.9426 | <0.0001 |
| Furosemide (at discharge) | 1.0756 | 1.0002 | 1.3287 | 0.0498 |
| Synthax score | 1.0353 | 1.0043 | 1.0658 | 0.0265 |
| LVEF < 45% | 1.3001 | 1.0260 | 1.6475 | 0.0179 |
| LAD PCI | 0.7001 | 0.5441 | 0.9009 | 0.0055 |
| Killip class (each class) | 3,1127 | 1,2362 | 6,1316 | <0.0001 |
| Number of stents (index ACS hospitalization) | 1.2131 | 1.0848 | 1.3567 | 0.0007 |
| STEMI vs NSTEMI (index ACS) | 0.8253 | 0.6892 | 0.9882 | 0.0366 |
| Nonfatal MACE (in-hospital index ACS) | 11.374 | 8.2310 | 14.639 | <0.001 |
| CABG (index ACS) | 0.8499 | 0.6625 | 0.9801 | 0.0203 |
